# Novel Activated Carbon Nanofibers Composited with Cost-Effective Graphene-Based Materials for Enhanced Adsorption Performance toward Methane

**DOI:** 10.3390/polym12092064

**Published:** 2020-09-10

**Authors:** Faten Ermala Che Othman, Norhaniza Yusof, Noorfidza Yub Harun, Muhammad Roil Bilad, Juhana Jaafar, Farhana Aziz, Wan Norharyati Wan Salleh, Ahmad Fauzi Ismail

**Affiliations:** 1 Advanced Membrane Technology Research Center (AMTEC), School of Chemical and Energy Engineering (SCEE), Universiti Teknologi Malaysia (UTM), Johor 81310, Malaysia; fermala2@live.utm.my (F.E.C.O.); juhana@petroleum.utm.my (J.J.); farhana@petroleum.utm.my (F.A.); hayati@petroleum.utm.my (W.N.W.S.); afauzi@utm.my (A.F.I.); 2 Department of Chemical Engineering, Universiti Teknologi Petronas (UTP), Bandar Seri Iskandar, Perak 32610, Malaysia; noorfidza.yub@utp.edu.my (N.Y.H.); mroil.bilad@utp.edu.my (M.R.B.)

**Keywords:** activated carbon nanofibers, graphene-based materials, graphene-derived rice husk ashes, composite adsorbent materials, carbon dioxide adsorption

## Abstract

Various types of activated carbon nanofibers’ (ACNFs) composites have been extensively studied and reported recently due to their extraordinary properties and applications. This study reports the fabrication and assessments of ACNFs incorporated with graphene-based materials, known as gACNFs, via simple electrospinning and subsequent physical activation process. TGA analysis proved graphene-derived rice husk ashes (GRHA)/ACNFs possess twice the carbon yield and thermally stable properties compared to other samples. Raman spectra, XRD, and FTIR analyses explained the chemical structures in all resultant gACNFs samples. The SEM and EDX results revealed the average fiber diameters of the gACNFs, ranging from 250 to 400 nm, and the successful incorporation of both GRHA and reduced graphene oxide (rGO) into the ACNFs’ structures. The results revealed that ACNFs incorporated with GRHA possesses the highest specific surface area (SSA), of 384 m^2^/g, with high micropore volume, of 0.1580 cm^3^/g, which is up to 88% of the total pore volume. The GRHA/ACNF was found to be a better adsorbent for CH_4_ compared to pristine ACNFs and reduced graphene oxide (rGO/ACNF) as it showed sorption up to 66.40 mmol/g at 25 °C and 12 bar. The sorption capacity of the GRHA/ACNF was impressively higher than earlier reported studies on ACNFs and ACNF composites. Interestingly, the CH_4_ adsorption of all ACNF samples obeyed the pseudo-second-order kinetic model at low pressure (4 bar), indicating the chemisorption behaviors. However, it obeyed the pseudo-first order at higher pressures (8 and 12 bar), indicating the physisorption behaviors. These results correspond to the textural properties that describe that the high adsorption capacity of CH_4_ at high pressure is mainly dependent upon the specific surface area (SSA), pore size distribution, and the suitable range of pore size.

## 1. Introduction

Fossil-based fuels are still the most dominant fuel for vehicles. Their combustion releases harmful by-product gases like oxides of sulfur and nitrogen [1], smoke, and particulate matters as well as carbon monoxide [2]. Carbon dioxide as the main combustion product is the primary reason for greenhouse gases’ effect and global warming [3]. Therefore, numerous efforts on development of alternative fuels have been borne at the local, regional, national, and global levels. 

Alternative fuels, such as natural gas (NG), especially methane (CH_4_), have been widely utilized mainly because they do not emit sulfur, mercury, or particulates and are considered much cleaner than other popular fossil fuels [4]. NG is considered as a better alternative due to its abundance as well as its cost-effective management. For usage, the CH_4_ must be in the form of either liquefied natural gas vehicles (LNGVs) or compressed natural gas vehicles (CNGVs). Both CNGVs and LNGVs thus require high compression tanks and the liquefaction occurs at cryogenic temperature, which can be costly and risky (requiring extensive safety precautions) [5]. In order to overcome this problem, adsorbed natural gas (ANG) has extensively been developed [6]. This method implements adsorbent materials that adsorb CH_4_ onto their surface under low pressure and ambient temperature. Early reports suggest that ANG is safer and more economical than CNGV or LNGV [7,8]. Most importantly, the gas adsorption and desorption are a reversible process, which is valuable for industrial applications. Various types of adsorbents have recently been proposed for CH_4_ adsorption. They are silica, zeolites, activated carbon (AC), metal-organic frameworks (MOFs), and clays. 

Granular or powdered AC offers the greatest adsorbent potential due to its high bulk density and high adsorption capacity [9] and is, hence, most commonly used [10]. However, despite posing large surface, AC lacks micropore volume, which could limit its adsorption capacity [11]. Meanwhile, newly modified AC in fibrous form has also been developed, known as AC nanofibers (ACNFs). The fibril structures in the ACNFs enhances the adsorption capacity, thanks to more accessible micropores from their external surface than the granular form [12]. High accessibility is important because the gas adsorbate must diffuse throughout the macropores and mesopores/micropores before reaching the adsorption sites, located deep inside the AC. Fibrous structures offer almost no diffusive resistance for adsorbate to reach the sites because of the absence of the macropores or mesopores network. Although the recently developed ACNFs have steadily overcome the drawbacks of the commercial AC, recent findings showed that pristine ACNFs possessed smaller surface area and lower micropore volume, which can be enhanced via incorporation of nanofillers/additives [13]. 

Graphene has been considered as the most promising additive due to its large theoretical specific surface area (SSA) and good electrical and thermal conductivities [14]. However, application of graphene is highly limited by its high cost of its precursor, complex synthesis method, and difficult fabrication scale-up. Accordingly, it leads to exploration of cheaper precursors (carbon-rich natural materials) and simpler synthesis methods. For example, biomass- and agricultural waste- (i.e., rice husk) based graphene has attracted major attention due to their abundant availability and cost-effectiveness. Recently, rice husks have been used to synthesize low-cost graphene via simple and scalable method for wide variety applications [15]. 

Study on incorporation of composite materials into ACNFs, especially graphene-based materials for CH_4_ adsorption, is still lacking. Hence, the main goal of this study was to develop and characterize graphene-modified ACNFs for CH_4_ adsorption. The performance of the resultant ACNFs was verified by using a volumetric adsorption system. 

## 2. Materials and Methods 

Polyacrylonitrile (PAN; molecular weight of 150,000 kDa) and N, N-dimethylformamide (DMF; 99.999%) were purchased from Sigma-Aldrich and were used without further modification or purification. Meanwhile, raw rice husk ashes and graphite powder were used to produce graphene-derived rice husk ashes (GRHA) and reduced graphene oxide (rGO) through a method proposed by Singh et al. (2017) [16] and Oliveira et al. (2018) [17], respectively. Other chemicals: Potassium hydroxide (KOH; ≥85% pellets), graphite powder, concentrated sulphuric acid (H_2_SO_4_), sodium nitrate (NaNO_3_), potassium permanganate (KMnO_4_), hydrogen peroxide (H_2_O_2_), and hydrochloric acid (HCl) were acquired from local supplier, VNK Supply & Services. Purified air (99.999%), nitrogen (N_2_; 99.999%), carbon dioxide (CO_2_; 99.999%), and methane (CH_4_; 99.999%) gases were purchased from Alpha Gas Solution Sdn. Bhd. 

### 2.1. Graphene Preparation from Rice Husk Ash

Rice husk ashes (RHA) were produced by heat treating the rice husk under air environment at 200 °C, followed by grinding for several minutes to form powder. The transformation of RHA into graphene-based structure was done using chemical activation method [16]. In this method, 1:5 ratio of RHA:KOH was placed compactly in a porcelain crucible, covered with a ceramic wool. The crucible was then put into a larger graphite crucible by covering the top with carbon powder and ceramic wool (1:1) to prevent the oxidation during high-temperature treatment. Subsequently, the RHA sample was annealed at 850 °C with heating rate of 5 °C/min under air environment. Later, deionized (DI) water was used to wash the resultant RHA for several times to remove the excess KOH and other impurities. The sample was then centrifuged and sonicated to obtain the supernatant. The obtained supernatant was then filtered using vacuum filter and left to dry overnight in an oven at 80 °C. The graphene-derived RHA obtained are known as GRHA [18].

### 2.2. Synthesis of Reduced Graphene Oxide (rGO)

Natural graphite powder was used as the precursor in the synthesis of graphene oxide (GO) through Hummer’s method [19]. In brief, 150 mL of H_2_SO_4_ (95–98%) was added into the mixture of graphite powder and NaNO_3_ (1/1, weight/weight ratio. The solution was stirred at temperature below 20 °C in an ice bath. Then, 18 g of KMnO_4_ were slowly added into the solution also under low temperature. After that, the temperature of the solution was slowly increased. As the temperature reached 35 °C, the mixture was then stirred for another 30 min. Then, DI water (300 mL) was added to form a yellowish-brown solution. Subsequently, the beaker was removed from the ice bath and the temperature of the solution was slowly increased to 98 °C and the mixture was stirred again overnight. Next, 300 mL of 30% H_2_O_2_ were introduced into the mixture. After yellow color bubbles appeared in the solution, 5% of HCl (1000 mL) was subsequently added in order to remove the metal ions and acid. The solution was later washed with DI water for several times until a neutral pH was achieved. The suspension was filtrated via vacuum filtration and the obtained GO was further dried under vacuum at 50 °C for 24 h. The GO sample was activated by using CO_2_ at 900 °C. Finally, the thermal reduction method by Zhao et al. (2010) [20] qA conducted in order to attain the (rGO).

### 2.3. Fabrication of Activated Carbon Nanofibers’ Nanocomposites (gACNFs)

Fifty mL of dope solution of 8 weight percent (w%) PAN in DMF were used to produce nanofibers (NFs) through electrospinning. Prior to electrospinning of NFs’ composite, 1 w% GRHA (relative to the polymer weight) was first dispersed in DMF and left for simultaneous stirring and sonicating for a few hours under room temperature. Then, PAN was added into the solution and was continuously stirred for another 24 h to obtain a homogenous solution. The same method was repeated for rGO/ACNF composite by excluding the addition of GRHA or rGO for pristine NFs.

### 2.4. Electrospinning and Pyrolysis of Nanofibers

The applied electrospinning parameters were obtained from various previous works [21]. In brief, the injection flow rate was 1.0 mL/hour, the high-voltage power supply was 10 kV, and the distance between the tip of the needle and collector was 15 cm. Furthermore, the chamber condition was set at 50% relative humidity (RH) and 32.5 °C [22]. The pristine NFs were denoted as NF, composite NFs with GRHA, and rGO were denoted as GRHA/NF and rGO/NF, respectively. The electrospun NFs were subjected to three stages of pyrolysis process to produce ACNFs. It started with thermal stabilization (oxidation), carbonization, and activation. Prior to heating, the NFs’ samples were placed in the porcelain combustion boat and then put inside the horizontal quartz tubular furnace (Carbolite CTF 12/65/550 with Eurotherm 2416 CC temperature control system). The stabilization was started from room temperature until 275 °C under the flow of air at heating rate of 2 °C/min. Then, the stabilized NFs were further carbonized until 600 °C under N_2_ atmosphere at heating rate of 5 °C/min and were physically activated with CO_2_ until 700 °C at heating rate of 5 °C/min. The resting time and gas flow rate were fixed at 30 min and 0.2 L/min, respectively, throughout the pyrolysis process. The fabrication parameters of all samples are summarized in Table 1.

### 2.5. Characterizations

The thermal behavior of the samples was analyzed using thermogravimetric analysis (TGA) under nitrogen atmosphere with heating rate of 10 °C/min at range of 50–700 °C (TG analyzer with differential scanning calorimeter (DSC; model STA8000). The structural variation of the ACNFs’ samples was identified by using Raman spectrometer (RAMAN plus Nanophoton). X-ray diffraction (XRD, Rigaku SmartLab) analysis was performed using Cu Kα (λ=1.54184 Å) at scanning rate of 1.5 ^o^/min. The IR spectra of the ACNFs were obtained by pressing the powdered ACNFs into potassium bromide (KBr) pellets using Fourier-transform infrared (FTIR, Thermo Scientific/Nicolet iS10) analysis with scanning range of 4000–1000 cm^−1^. The diameter and morphology of as-prepared ACNF samples were analyzed using scanning electron microscope (SEM; JSM 6701-F, JEOL, Japan) equipped with electron dispersive X-ray (EDX; Hitachi Co. Ltd., Japan) to determine the elemental mapping of the samples.

Prior to N_2_ adsorption measurements, the ACNFs were first degassed in a processor at 350 °C under vacuum 1 × 10^−1^ kilopascal (kPa) for 3 h. After pretreatment, pore texture characterizations were carried in a porosity analyzer MicrotracBEL Belsorp-max with N_2_ (99.9999% purity) at a temperature of −196 °C for adsorption–desorption experiments. According to the data, the SSA, total pore volume, and mean pore diameter of ACNFs were calculated by the Brunauer-Emmett-Teller (BET) method. The micropore surface area and micropore volume of ACNFs were determined by the t-plot and Barrett-Joyner-Halenda (BJH) methods, respectively, according to the BELSORP analysis program software. All characterizations of SSA, pore volume, and pore size distribution of the resulting ACNF samples from N_2_ adsorption–desorption measurements were performed in at least triplicate. 

### 2.6. Methane Adsorption Performance via Volumetric Method

The 0.3 g of each ACNF sample was weighed and dried in a vacuum oven for 24 h at 150 °C. After completely drying, the ACNFs were weighed again and further loaded into the adsorption cell, detailed in previous work [23]. Meanwhile, in the loading cell, CH_4_ was injected until reaching desired pressures (4, 8, and 12 bar). To start the adsorption test, the valve between the adsorption and loading cells was opened to let the CH_4_ from the loading cell pass through the ACNFs located in the adsorption cells. The pressure changes in both cells were recorded continuously at 5-min intervals until the equilibrium pressure was achieved, indicated by constant pressure reading for about 10 min. The adsorbed amount of CH_4_ was calculated according to Nasri et al. (2014) by using Equation (1).

(1) $$q=\frac{1}{m}\left[\frac{Vv}{R}\left(\left|\frac{P}{ZT}\right|i-\left|\frac{P}{ZT}\right|eq\right)a+\left(\left|\frac{P}{ZT}\right|i-\left|\frac{P}{ZT}\right|eq\right)l\right]$$

where *q* is the amount of CH_4_ adsorbed, *m* is the mass of the adsorbents (g), *V* is the volume (cm^3^), *R* is the gas constant, *P* is the pressure (bar), *T* is temperature (K), *a* is adsorption cell, *l* is loading cell, *i* is initial state, *eq* represents the equilibrium state of the final adsorption, and *Z* is the compressibility factor.

### 2.7. Adsorption Kinetics

The adsorption of CO_2_ onto the ACNFs was modeled by using pseudo-first- or pseudo-second-order kinetic as in Equations (2) and (4), respectively.

(2) $$\frac{d_{qt}}{dt}=k_{1}\;{\left(q_{e}-q_{t}\right)}^{2}$$

Equation (2) can then be rewritten into linear form, as in Equation (3).

(3) $$\ln\left(q_{e}-q_{t}\right)=lnq_{e}-kt$$

(4) $$\frac{d_{qt}}{dt}=k_{1}{\left(q_{e}-q_{t}\right)}^{2}$$

Equation (4) can then be rewritten into linear form, as in Equation (5).

(5) $$\frac{t}{q_{t}}=\frac{1}{k_{2}q_{e}^{2}}-\frac{t}{q_{e}}$$

where $q_{t}$ is the amount of adsorbed CH_4_ at any time (mmol/g), $q_{e}$ is the amount of adsorbed CH_4_ at equilibrium (mmol/g), and $k_{1}$ and $k_{2}$ are rate constant for pseudo-first- and pseudo-second-order model, respectively. 

## 3. Results and Discussion

### 3.1. Physicochemical Properties of the gACNFs

**Figure 1:**
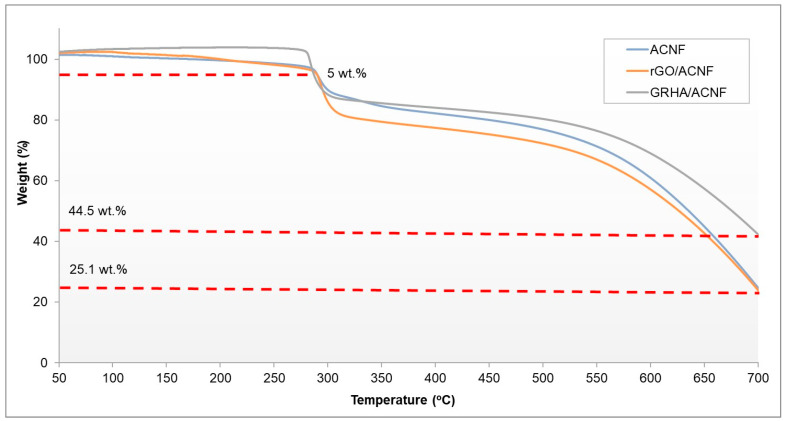
TGA thermogram of the samples. ACNF: activated carbon nanofibers, rGO: ACNF: activated carbon nanofibers, GRHA: graphene-derived rice husk ashes.

TGA thermograms of the pristine and the composite NFs are shown in Figure 1. All samples show two stages of decompositions. The first stage (~5 wt.%) occurred at 285–320 °C and slowed down at 340–550 °C. The first-stage weight loss can be ascribed to the decomposition of inorganic components and loss of moisture of the PAN polymer [24,25]. PAN-based NFs were found to degrade at a slightly lower temperature (95 to 120 °C) [26]. However, in this study, the degradation started at a higher temperature (285 °C), most likely because of the cross-linking of PAN chains forming an aromatic ladder structure to avoid melting of NFs, as reported earlier [27]. Formation of stable 3D cyclized cyano groups’ structure in the chain segments of the PAN polymer was also possible during the cross-linking in the oxidative atmosphere at lower temperature (200–300 °C) [28].

The second stage of weight loss starts around 500 °C, with a dramatic weight loss (>50%) when the temperature gradually increased up to 700 °C. At 700 °C, both pristine ACNF and rGO/ACNF exhibited similar carbon yield, which was ~25.1 wt.%, while the yield for GRHA/ACNF was ~44.5 wt.%, almost twice the others’. The high yield of the GRHA/ACNF was possibly due to the presence of silica that improved the thermal stability [29,30]. The second stage of degradation can also be ascribed to further aromatization of the formed cyclic structures. At higher temperatures, above 700 °C, hydrogen was evolved and the rings became aromatic [26,31]. 

**Figure 2:**
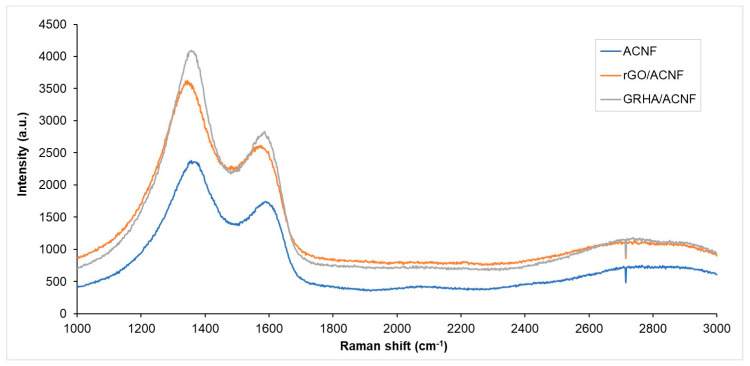
Raman spectra of the samples. ACNF: activated carbon nanofibers, rGO: ACNF: activated carbon nanofibers, GRHA: graphene-derived rice husk ashes.

Raman spectra of the pristine ACNFs and modified ACNFs are presented in Figure 2. In Raman spectra, there are three major important bands, known as D, G, and 2D bands, to determine the crystallinity of the graphite-based materials. From the spectra in Figure 2, the most prominent peaks can be observed at 1350, 1590, and 2680 cm^-1^ in all samples, which represent D, G, and 2D band, respectively [32,33]. All samples exhibited high D and G band and extremely broad 2D band. The presence of D band in the spectra was attributed to the existence of disordered carbonaceous structure, while the G band indicated the presence of ordered graphitic structure [34]. Meanwhile, 2D band was produced due to phonon-scattering process, also associated with the presence of graphene layers in materials [35]. 

The D band was higher than the G band, indicating more disordered structures in the ACNFs (Figure 2). This result is supported with the “R-value”, or intensity ratio, of the samples. The smaller the “R-value”, the more ordered graphite crystallites are [34]. The R-values of the pristine ACNFs, rGO/ACNF, and GRHA/ACNF were 1.17, 1.40, and 3.17, respectively, which indicated that the addition of rGO or GRHA promoted the formation of more disordered or defective graphitic structures in the ACNFs. According to Liu and Wilcox (2011) [36], the gas adsorbates showed stronger binding interactions with the defective site on the surface of adsorbents as compared to the surface of perfect adsorbents.

**Figure 3:**
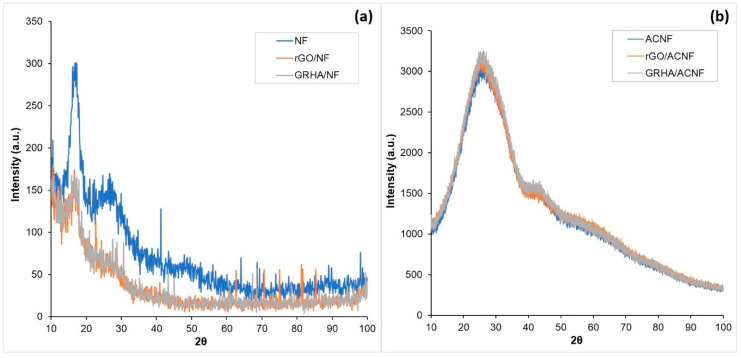
XRD spectra of pristine and composite nanofibers (**a**) prior to activation and (**b**) after activation. ACNF: activated carbon nanofibers, rGO: ACNF: activated carbon nanofibers, GRHA: graphene-derived rice husk ashes.

Figure 3 shows the XRD spectra of the NFs prior to and after activation of ACNFs. It shows the materials containing random microcrystalline carbon fragments are in amorphous forms, possibly due to the existence of various inorganic compounds and impurities. However, there are two distinct broad peaks at 17.6^o^ and 28^o^ in all samples prior to activation, most likely corresponding to the crystallographic planes (100) and semi-crystalline PAN (110) [37,38]. After activation, the spectra exhibit very broad diffraction peaks with the absence of a sharp peak. This result reveals that all the resultant ACNFs were predominantly amorphous. The spectra showed one major, high, and broad peak at 26^o^ and another weak, broad peak at 43^o^. In comparison to the study conducted by Dong et al. (2014) [39], they detected the crystalline graphite peak at 2θ = 28^o^, and this slightly shifted to the left peak, obtained in the present study, indicating the enlargement of the distance between the graphene layers. These two peaks at 26^o^ and 43^o^ correspond to the crystallographic planes of (002) and (100) in graphitic structures, respectively. A shoulder at 43^o^ in all resultant ACNFs indicates the absence of a repetitively stacked graphitic structure [40].

**Figure 4:**
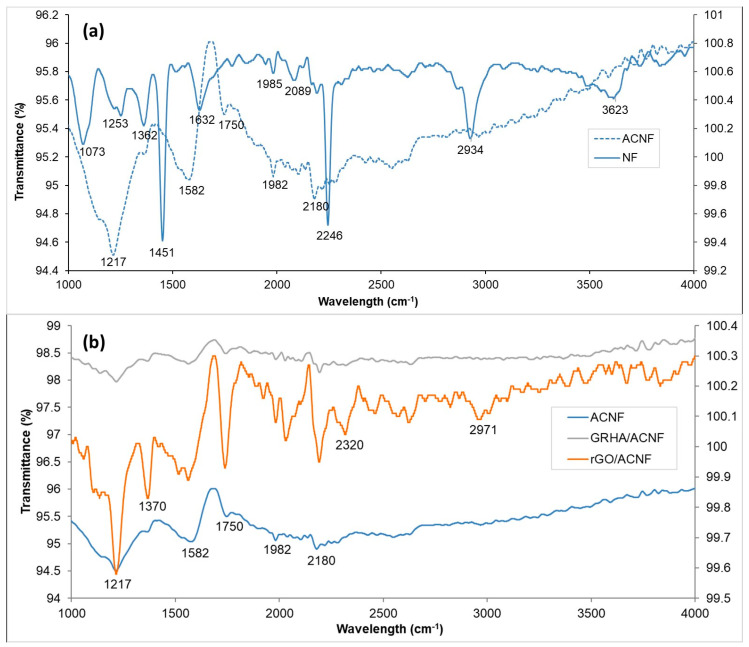
FTIR spectra of pristine and composite nanofibers (**a**) prior to activation and (**b**) after activation. ACNF: activated carbon nanofibers, rGO: ACNF: activated carbon nanofibers, GRHA: graphene-derived rice husk ashes.

The chemical structures of all NFs (pristine and modified) prior to and after activation were confirmed with FTIR. The FTIR spectra of pristine NFs prior to and after activation are revealed in Figure 4a. Prior to activation, there were 10 peaks that can be observed at 1073, 1253, 1362, 1451, 1632, 1985, 2089, 2246, 2934, and 3623 cm^−1^. The peaks appeared in the range of 1000–2000 cm^−1^ and are attributed to the bending and stretching of C–H, O–H, and C–C of PAN. The band at 2000–3000 cm^−1^ shows the presence of alkynes (C≡C), nitrile groups (C≡N) [28], and alkanes’ stretch (C–H) [41,42]. Moreover, the presence of the asymmetric bending and stretching vibration of surface hydroxyls and adsorbed water was indicated by the appearance of band at 3200–3600 cm^−1^ [43]. However, most of the described peaks disappeared due to the decomposition of PAN and removal of transition compounds during the high activation temperature, leaving only carbon and hydrogen bonds at 1217, 1582, 1750, 1982, and 2180 cm^−1^, as shown in Figure 4a. 

Figure 4b reveals the FTIR spectra of pristine ACNFs, rGO/ACNFs, and GRHA/ACNF after activation. All three samples exhibited similar peaks but with different intensities. The pristine ACNFs exhibited the highest intensities. The appearance of peaks at 1217, 1582, 1750, 1982, and 2180 cm^−1^ in the spectrum verifies the existence of C–O stretching vibrations of epoxide groups, aromatic –C=C– bonds, C=O stretching, and alkynes’ (C≡C) stretches, respectively [44]. The disappearance of C≡N after the activation indicates the production of ring structures in PAN-based ACNFs [24]. As both applied additives were carbon-based materials, there was no “extra” peak observed unless the appearance of a weak and small peak of asymmetric stretches of Si-O-Si at 1040 cm^−1^ [45] due to the presence of silica in the GRHA samples. These obtained results correspond to the EDX analysis, as it confirmed the existence of C and O in all samples with different percentages.

### 3.2. Morphologies and Structures

**Figure 5:**
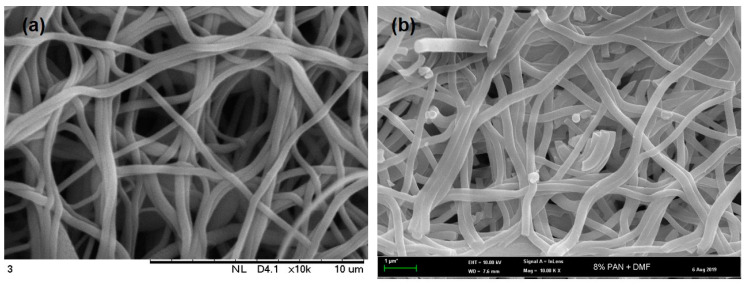
The morphology of pristine nanofibers (**a**) prior to activation and (**b**) after activation.

**Figure 6:**
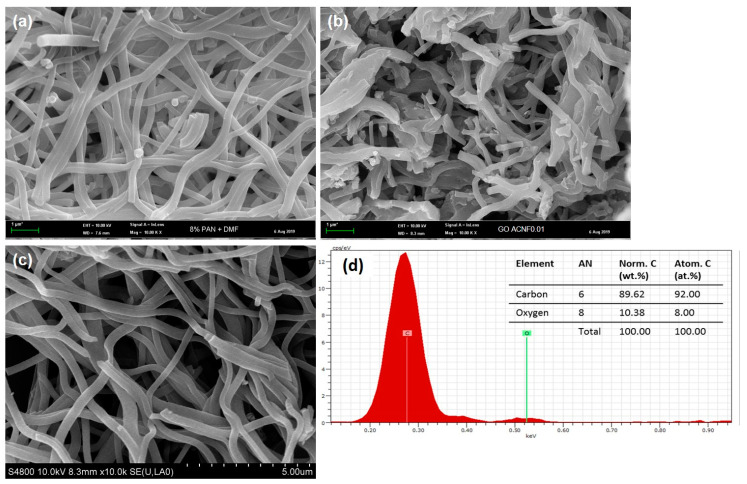
The morphology of (**a**) pristine activated carbon nanofibers (ACNFs), and ACNFs with different graphene-based additives, (**b**) with reduced graphene oxide (rGO), (**c**) with graphene-derived rice huskashes (GRHA), and (**d**) EDX mapping of rGO/ACNF.

The morphologies of all resultant NFs are shown in Figure 5 and Figure 6. Most of the NFs were stuck with each other, forming an interconnected/fused fibrous structure with a wide range of diameter. It is believed that the formation of the fused fibrous structure could be due to the insufficient solvent evaporation from the polymer jets [46]. Yet, this structure showed insignificant effect to its performance. As these resultant NFs were further carbonized, the fiber diameter was reduced, resulting in high surface area. The changes in porous characteristics and surface area of the NFs had significant effects on gas adsorption, as detailed later. 

Figure 5 shows the morphology of the NFs prior to and after activation. Prior to activation, the NFs exhibited smooth, straight, and almost aligned structure with a minimum amount of beads. The average diameter of the NFs ranged from 400–550 nm. After activation at 800 °C, the structure of the NFs became coarser and wrinkled, with the appearance of several beads. The fiber diameter also shrank to 300–500 nm, due to the vulnerability of the surface toward the heat treatment (loss of water content) and breakage of the hydrogen bonds at increasing temperature, as reported earlier [47]. Moreover, the addition of either rGO or GRHA into the NFs further decreased diameters, to 250–400 nm (up to 50%). This is because the properties of graphene with high conductivity would affect the properties of dope solution, including the electrical conductivity, which had a major impact on the fibers’ diameter [33]. Even though the range of the fiber diameter obtained was not in nanoscale, which is <100 nm, NFs’ term has been used throughout this study, referring to the incorporation of nanomaterials, such as GRHA and rGO, to produce NFs’ composites. 

Figure 6 shows the microstructure morphologies of pristine and composite ACNFs (rGO/ACNFs and GRHA/ACNFs) after activation. No major change was observed on morphologies of either composite ACNFs as compared to its original pristine ACNFs (coarser and wrinkled). However, it slightly affected the diameter of the ACNFs, in which the composite ACNFs possessed a smaller diameter. Surprisingly, the composite ACNFs, in Figure 6b,c, exhibited a beadless structure, an observation for the first time reported in literature. A smooth structure with no beads or agglomeration is needed in order to obtain ACNFs with high SSA as there was no bead that blocked the surface area during the adsorption process. The mean diameter of rGO/ACNFs and GRHA/ACNFs ranged between 300 to 500 nm and 200–350 nm, respectively. The existence of each element in the resultant ACNFs was confirmed with EDX analysis. Figure 6d shows the EDX mapping of rGO/ACNF with 92 atomic percent (at.%) of carbon and 8 at.% of oxygen. Because rGO (carbon-based materials) was used as additive, there were no other elements or impurities detected. Meanwhile, for GRHA/ACNF, the EDX mapping obtained from our preliminary studies, as previously reported by Othman et al., was used for comparison with rGO/ACNF. From their report, it can be observed that the GRHA/ACNF composites possessed three important elements in their structures, which were 94.19 at.% of carbon, 5.43 at.% of oxygen, and 0.38 at.% of silicon [48]. There was still a small amount of silicon observed in the structure, as this proved the existence of the silica in the GRHA derived from the rice husk ashes (RHA). 

### 3.3. Pore Structure and Texture of gACNFs

**Figure 7:**
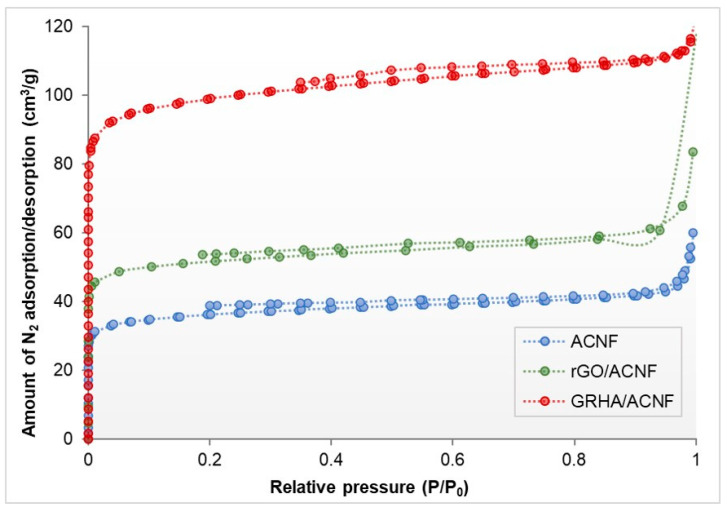
Nitrogen adsorption/desorption of pristine ACNF, rGO/ACNFs, and GRHA/ACNF at −196 °C and 1 bar. ACNF: activated carbon nanofibers, rGO: ACNF: activated carbon nanofibers, GRHA: graphene-derived rice husk ashes.

Figure 7 shows the SSA and the porous structure behavior of all resultant ACNFs determined by nitrogen (N_2_) adsorption isotherms. The sharp adsorption of N_2_ curve at low pressure of <0.1 bar. indicates the micropore filling and monolayer adsorption phase [49]. As the pressure increased over 0.1, the isotherms became nearly plateau (ranging from 0.15–0.95), which was due to the multilayer adsorption on the mesopores of the ACNFs. However, as the saturation pressure approached, a significant improvement on N_2_ adsorption is observed between pristine ACNFs and composite ACNFs, which increased from 60 cm^3^/g up to 84 cm^3^/g and 117 cm^3^/g for rGO/ACNF and GRHA/ACNF, respectively. To some extents, the adsorption isotherms of those three ACNF samples (ACNF, rGO/ACNF, and GRHA/ACNFs) were identical, which were the combination of both Type I and Type IV, indicating the presence of micropores and mesopores [25,50]. Even though all the plotted curves exhibit similar characteristics, the quantity of N_2_ adsorbed varied in each sample, denoting the pore structures’ variations. Interestingly, the adsorbed amount of N_2_ obtained by GRHA/ACNFs was twice the value of the pristine ACNF and slightly higher than the rGO/ACNFs. These findings are in agreement with the SSA results (will be discussed later). 

Table 2 summarizes the porous structure parameters, including SSA, total pore volume (TPV), micropore volume (V_micro_), and average pore diameter (DP_ave_) of pristine and composite ACNFs prior to and after activation. It shows that activation increased the SSA of all ACNFs dramatically, thanks to the creation of new micropores’ structures [51]. There was no significant increment in the SSA in all composite NF samples prior to activation. However, the value of the SSA was twice the SSA value of the pristine ACNFs after the physical activation. Prior to activation, rGO/ACNF exhibited the smallest DP_Ave_ value. However, the value was the largest after activation, as shown in Table 2. This was probably due to the fast decomposition of rGO during carbonization (around 300–650 °C) (Figure 1), which minimized the catalytic effect of rGO during activation process, as its decomposition was getting slower, >650 °C, producing larger DP_Ave_ compared to other samples. In this study, it was believed the minimum temperature for catalytic effect of rGO to take place is >700 °C, in order to produce maximum micropores and pore diameter reduction. 

Table 2 shows that both GRHA/NF and GRHA/ACNF exhibited the highest SSA increments from 17.8035 m^2^/g and 384.65 m^2^/g, respectively, the highest among all the ACNFs. They correspond to the TPV and V_micro_ obtained. In gas adsorption, surface area as well as the wide range of porous structures (depending on the types and size of gas molecules), were the main performance-determining factors. Generally, adsorbent with high SSA and high pore volume is desirable [52]. In Table 2, GRHA/ACNF exhibited the highest SSA, TPV, and V_micro_ of 384.65 m^2^/g, 0.1785 cm^3^/g, and 0.1580 cm^3^/g, respectively. These results agree with the CH_4_ adsorption performances discussed later.

### 3.4. Adsorption Performance and Kinetic Study of gACNFs

**Figure 8:**
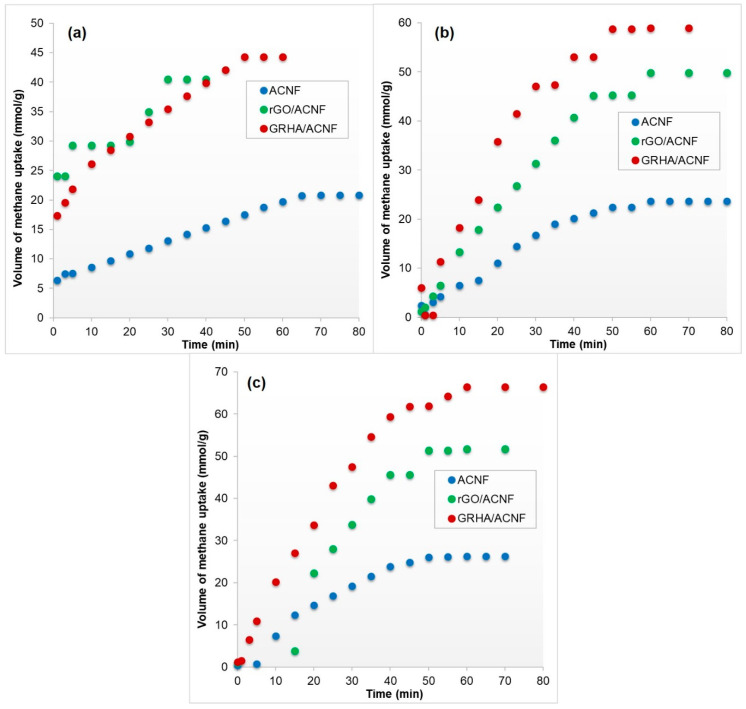
Adsorption profile of methane to reach equilibrium on the ACNF, rGO/ACNF, and GRHA/ACNF at different pressures: (**a**) 4 bar, (**b**) 8 bar, and (**c**) 12 bar. ACNF: activated carbon nanofibers, rGO: ACNF: activated carbon nanofibers, GRHA: graphene-derived rice husk ashes.

Figure 8 shows the CH_4_ adsorption performances of all ACNFs at different pressures. In Figure 8a, it can be seen that the GRHA/ACNF exhibits the highest CH_4_ adsorption capacity, of 44.32 mmol/g, followed by rGO/ACNF of 40.52 mmol/g and ACNF of 20.86 mmol/g at 4 bar. Meanwhile, Figure 8b,c exhibit the adsorption profile of CH_4_ at other pressures, which was 8 bar and 12 bar, respectively. With an increasing pressure, the CH_4_ adsorption capacity of all ACNF samples was gradually increased and reached a smooth value at equilibrium state. As expected, the adsorption performance of all ACNF samples showed similar trends as the one at lower pressure (4 bar), which was GRHA/ACNF>rGO/ACNF>ACNF.

These results correspond well with the N_2_ adsorption isotherms and the SSA (see Figure 7 and Table 2), in which high SSA was attributed to high adsorption capacity due to the physisorption [53]. This means that the adsorption of CH_4_ was mainly dependent upon the SSA, pore size distribution, and the ratio of the suitable pore sizes [54]. Interestingly, although the obtained SSA of GRHA/ACNF composite was lower than some references [55], as tabulated in Table 3, its adsorption performance towards CH_4_ was significantly higher, making this newly fabricated GRHA/ACNF composite a suitable candidate for good gas adsorbents. This is possibly due to the well distributed pore size distribution between the micropores (up to 90% of the TPV) and mesopores available in the entire ACNFs structures, which played significant role in the adsorbent-adsorbates interaction. The micropore size ranging from 1.3954 to 2.174 nm exhibited larger adsorption sites for CH_4_ molecules with size of 0.38 nm. and this made the CH_4_ adsorption onto the ACNFs surface much easier.

**Figure 9:**
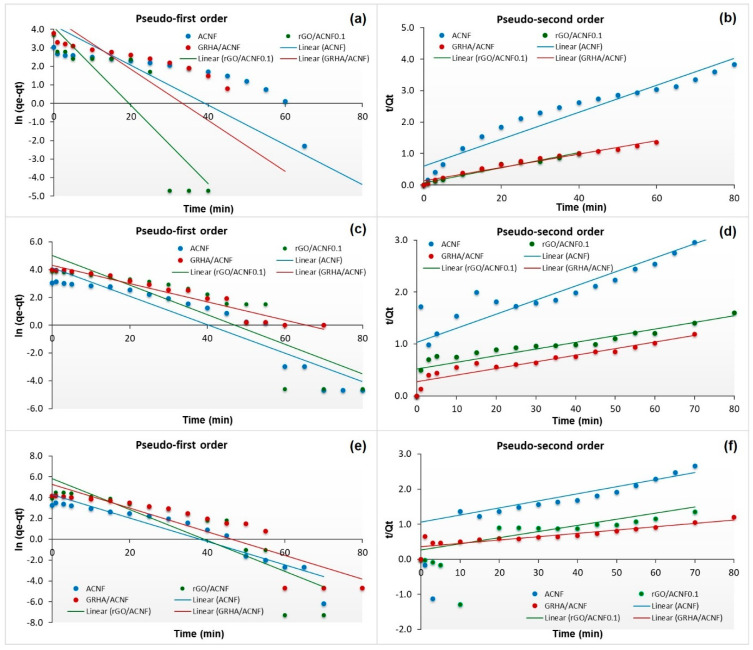
Kinetic adsorption of pseudo-first- and pseudo-second-order models of all ACNF samples at different pressures (**a**,**b**) 4 bar, (**c**,**d**) 8 bar, and (**e**,**f**) 12 bar.

Figure 9 shows the adsorption kinetic of all ACNF samples based on pseudo-first- and pseudo-second-order kinetic models at different pressures. As can be seen, the pseudo-second-order kinetic model exhibited greater value of all the coefficient correlations (R^2^) than the pseudo-first-order kinetic model, which were 0.9262, 0.9685, and 0.9737 for ACNF, rGO/ACNF, and GRHA/ACNF, respectively, at adsorption pressure of 4 bar. Among the samples, GRHA/ACNF possessed the highest R^2^ value, of 0.9737. It suggests that the adsorption of CH_4_ towards ACNFs obeyed the pseudo-second-order kinetic models, indicating that the sorption kinetics of CH_4_ occurred on the microporous structure of ACNFs involved in the chemisorption [60]. This result is in good agreement with the N_2_ adsorption isotherm and SSA data. This finding was supported by a previous study conducted by Tang and co-workers (2007) [61], as they also found that the ACNFs-based adsorbents obeyed the pseudo-second-order kinetic model. Interestingly, at higher pressures, of 8 and 12 bar, all samples seemed to obey the pseudo-first-order kinetic model, with higher R^2^ value than pseudo-second-order kinetic model, as tabulated in Table 4. R^2^ values of GRHA/ACNF at 8 and 12 bars were 0.9369 and 0.8054, respectively. This is believed due to the occurrence of physical adsorption because of the formation of multilayers of CH_4_ molecules on the heterogeneous surface of the ACNFs at higher adsorption pressure.

## 4. Conclusions

Incorporation of either GRHA or rGO showed great improvement in ACNF’s structure as well as its adsorption performance. The adsorption capacity was highly dependent upon the SSA and micropore volume as well as the pore size of the adsorbents; the higher the SSA and micropore volume, the higher the adsorption capacity. As expected, the CH_4_ uptakes showed similar trend to the SSA results as follows: GRHA/ACNF>rGO/ACNF>ACNF. The results revealed that the CH_4_ adsorption capacity by GRHA/ACNF was the highest, with value of 44.33 mmol/g, which is nearly double the volume of the pristine ACNFs, of 20.86 mmol/g, and slightly higher than rGO/ACNF, of 40.52 mmol/g, at 4 bar. Meanwhile, at 8 and 12 bar, the adsorption values were improved to 58.94 and 66.40 mmol/g, respectively. As the pressure increased, the adsorption capacity also increased. These adsorption values of all samples showed great improvement compared to previously reported ACNFs’ composites and this proved the resultant ACNFs with high heterogeneity surfaces as suitable adsorbents for CH_4_ adsorption and storage.

## Figures and Tables

**Table 1:** Dope formulation of different graphene precursors of 50-mL solution.

Activation	Sample Name	PAN to Graphene Ratio	PAN wt. (g)	Graphene wt. (g)	Graphene
Prior	NF	-	4	-	-
	GRHA/NF	100:1	4	0.04	GRHA
	rGO/NF	100:1	4	0.04	rGO
After	ACNF	-	4	-	-
	GRHA/ACNF	100:1	4	0.04	GRHA
	rGO/ACNF	100:1	4	0.04	rGO

**Table 2:** Porous structure characteristics of pristine and composite activated carbon nanofibers s prior to and after activation.

	Samples	SSA (m^2^/g)	TPV (cm^3^/g)	V_micro_ (cm^3^/g)	DP_Ave_ (nm)
Prior activation	NF	17.1723	0.1364	−0.0064 *	31.7692
	rGO/NF	10.2330	0.0737	−0.0008	28.8255
	GRHA/NF	17.8035	0.1423	−0.0072	31.9677
After activation	ACNF	137.0900	0.0807	0.0534	2.3559
	rGO/ACNF	205.3000	0.1665	0.0825	3.2884
	GRHA/ACNF	384.6500	0.1785	0.1580	1.8564

SSA = specific surface area; TPV = total pore volume; V_micro_ = micropore volume; DP_Ave_ = Average pore diameter. * Micropore volume in NFs prior to activation was negative due to the absence of micropores in the samples.

**Table 3:** Comparison of methane adsorption capacity on various activated carbon fibers and their composite-based adsorbents.

Materials	SSA (m^2^/g)	TPV (cm^3^/g)	Vol. of CH_4_ adsorbed (mmol/g)	Temp; Pressure	Ref.
ACNF	137	0.0807	20.84	25 °C; 4 bar	This work
rGO/ACNF	205	0.1665	40.52	25 °C; 4 bar	
GRHA/ACNF	384	0.1785	44.32	25 °C; 4 bar	
GRHA/ACNF	384	0.1785	58.94	25 °C; 8 bar	
GRHA/ACNF	384	0.1785	66.40	25 °C; 12 bar	
ACF-K_2_CO_3_	2500	0.8	191.3 V/V	25 °C; 35 bar	[56]
ACF	1965	0.41	7.40	25 °C; 40 bar	[57]
ACF-NH_3_	1795	1.0231	8.45	25 °C; 55 bar	[54]
ACF	1511	-	9.83 wt%	25 °C; 18 bar	[58]
MgO/ACNF	1893	0.6212	2.37	25 °C; 3.5 bar	[59]
MnO_2_/ACNF	431	0.1861	1.35	25 °C; 3.5 bar	
ACNF	478	0.2097	1.42	25 °C; 3.5 bar	

**Table 4:** Kinetic parameters of pseudo-first-order and pseudo-second-order for ACNF, rGO/ACNF, and GRHA/ACNF at different adsorption pressures.

Sample	Pressure (bar)	q_e_, _exp_ (mmol/g)	Pseudo-First Order		Pseudo-Second Order	
			*k*_1_	R^2^	*k*_2_	R^2^
ACNF	4	20.87	0.1382	0.7004	0.0791	0.9262
rGO/ACNF		40.52	0.2209	0.7679	0.3785	0.9685
GRHA/ACNF		44.33	0.1645	0.7299	0.1765	0.9737
ACNF	8	23.67	0.1102	0.8773	0.0408	0.8184
rGO/ACNF		49.88	0.1202	0.8577	0.0385	0.8048
GRHA/ACNF		58.94	0.1577	0.9369	0.0619	0.8780
ACNF	12	26.32	0.1495	0.8817	0.0358	0.0911
rGO/ACNF		51.76	0.1869	0.7677	0.0737	0.1325
GRHA/ACNF		66.40	0.1246	0.8054	0.0415	0.7875
